# Paget-Schroetter Syndrome: A Rare Case of Upper Extremity Deep Vein Thrombosis in a Young Swimmer

**DOI:** 10.7759/cureus.26060

**Published:** 2022-06-18

**Authors:** Amar Suwal, Swarup Sharma Rijal, Manish Shrestha, Salina Munankami, Sijan Basnet

**Affiliations:** 1 Internal Medicine, Tower Health Medical Group, Wyomissing, USA; 2 Internal Medicine, Reading Hospital, Tower Health, Reading, USA; 3 General Medicine, Kathmandu Medical College, Kathmandu, NPL

**Keywords:** therapeutic anticoagulation, first rib resection, thoracic outlet, upper extremity deep vein thrombosis, paget-schroetter syndrome

## Abstract

Paget-Schroetter syndrome (PSS) is a rare form of spontaneous upper extremity deep vein thrombosis associated with vigorous activity of the upper extremities. We present a rare case of a young swimmer who presented with a painful right upper extremity swelling, with compression ultrasound (CUS) revealing extensive venous clots in the basilic, axillary, and subclavian veins. Venous duplex revealed extrinsic compression of the subclavian vein, and catheter-based contrast venography confirmed our diagnosis of PSS. The patient was started on a therapeutic dose of subcutaneous enoxaparin and referred to a higher center for further intervention.

## Introduction

Paget-Schroetter syndrome (PSS) is a rare cause of spontaneous unilateral upper extremity deep venous thrombosis (UEDVT) [1,2], occurring in young athletic patients usually due to subclavian vein compression at the thoracic outlet, following repetitive over-the-head strenuous upper extremity activity [1,3,4]. With an annual incidence of one to two per 100,000 individuals [3]. PSS accounts for up to 20% of all cases of upper extremity DVTs [1]. Other predisposing causes of DVT, like any indwelling hardware (central line, pacemakers, and ports) and thrombophilic states (occult or overt malignancy), need to be excluded before considering PSS as a possible diagnosis [1]. Here, we report an interesting case of PSS in a young swimmer.

## Case presentation

A 32-year-old nonsmoker, fairly active lady, and an avid swimmer, without comorbidities, was initially seen in the emergency department (ED) one week ago and diagnosed with right UEDVT (axillary thrombus) and right lower lobe segmental pulmonary embolism (PE) after presenting with a day of painful swelling of the right upper extremity. It was thought to be provoked by oral contraceptives (OCP) with estrogen, and she was subsequently discharged on apixaban from the ED. Despite stopping the OCP and being religiously compliant with apixaban, she reported persistent symptoms for one week, thus prompting her second visit to the hospital. She denied significant trauma, immobilization, or major surgery to her affected arm. She usually goes swimming during her leisure time and recalled going for a swim on the day before the onset of symptoms. She denied a history of venous access to the right upper extremity, personal history of DVT, abortion, or miscarriages. She also denied a history of fever, insect bite, or rash. She was fully vaccinated for coronavirus disease 2019 (COVID-19), the last dose received three months back. She had no family history of bleeding disorders or clotting disorders, and she denied intravenous recreational drugs use.

On presentation, she had a temperature of 36.8°C, heart rate of 66 beats per minute, blood pressure of 125/95 mmHg, respiratory rate of 19 breaths per minute, and saturation of 96% on room air. On physical examination, she had tender right upper arm swelling with overlying erythema and a cord-like superficial vein extending from the lower cervical region to the elbow. She had an unremarkable neurological examination in the affected extremity, with intact distal pulses. The rest of the physical examination was normal.

**Figure 1:**
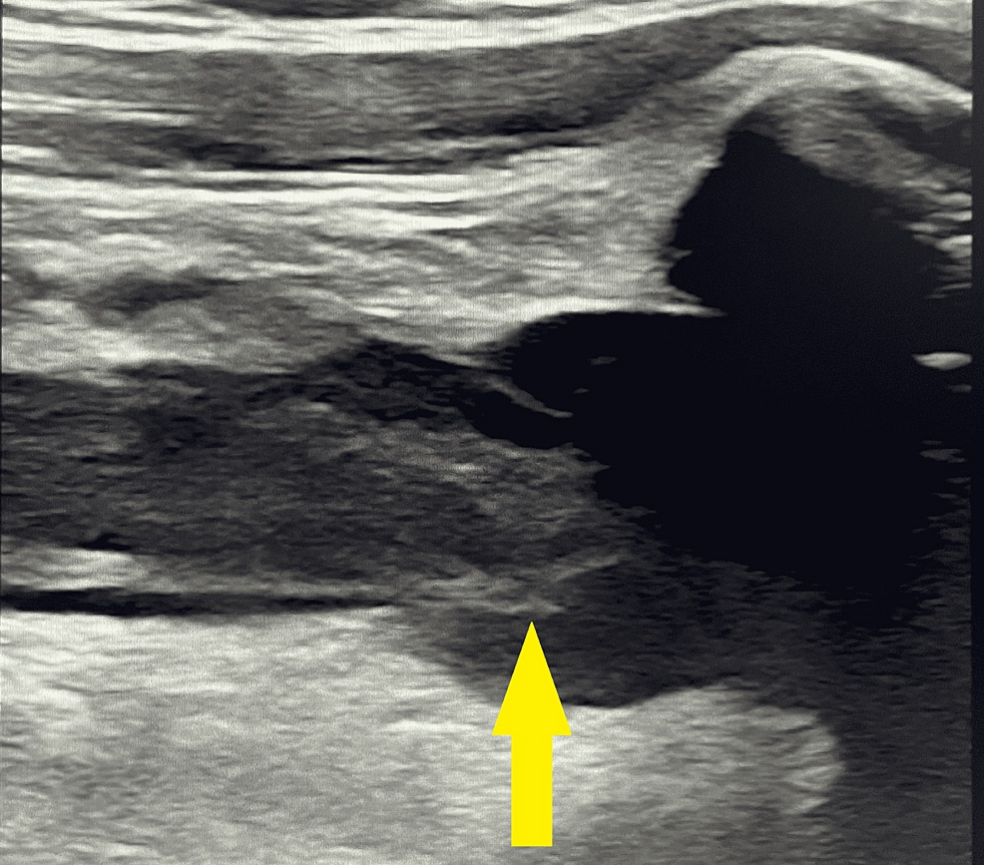
Ultrasound of right brachiocephalic vein with thrombus (arrow).

**Figure 2:**
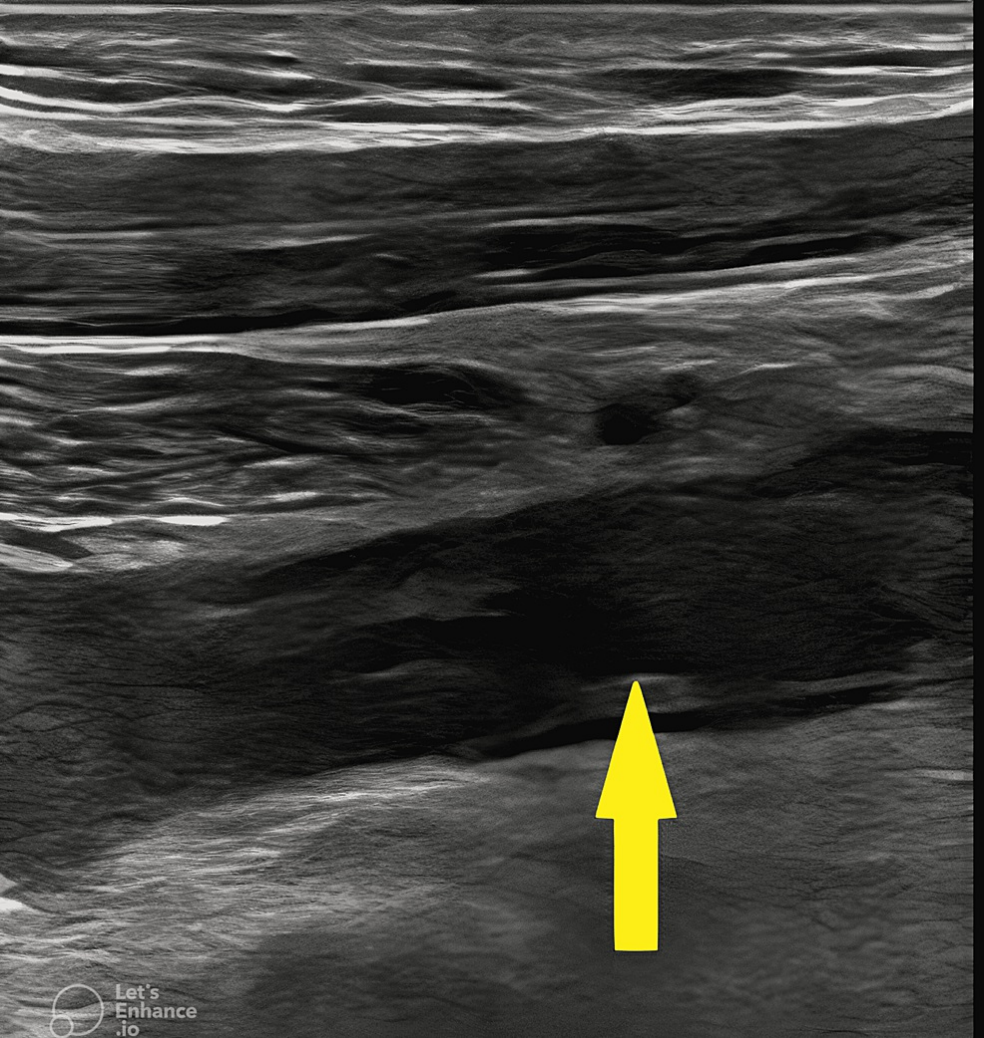
Ultrasound of right subclavian vein with thrombus (arrow).

**Figure 3:**
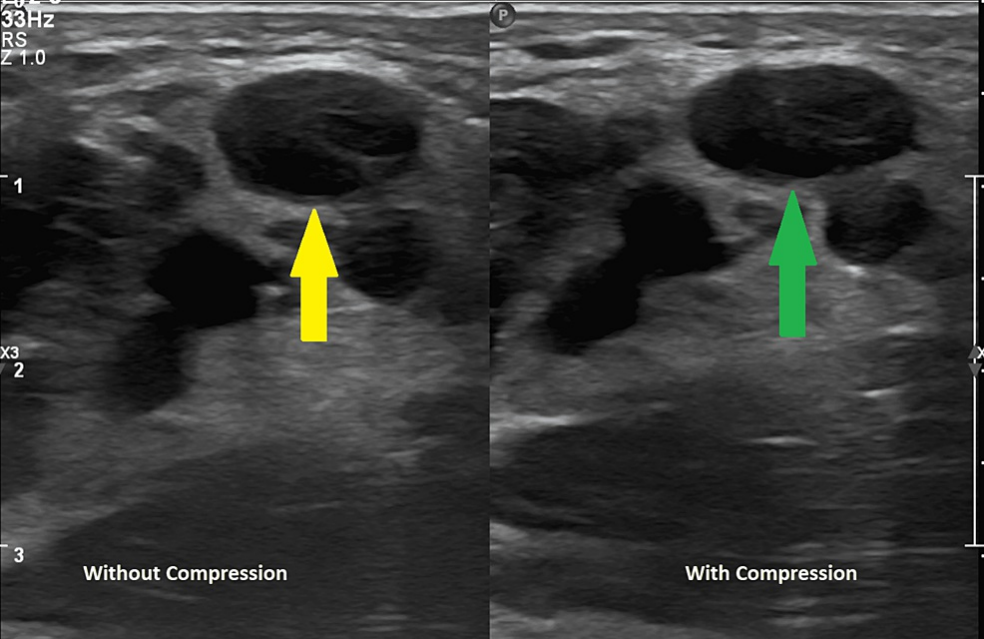
Ultrasound showing a non-compressible right axillary vein (yellow arrow: right axillary vein without compression; green arrow: with compression).

She underwent an extensive thrombophilia workup (Table 1). Flow cytometry for paroxysmal nocturnal hematuria was normal, and the COVID-19 test was negative. Compression ultrasound (CUS) in the ED showed extension of the venous clot from the basilic to the axillary vein into the subclavian vein (Figures 1-3).

**Table 1:** The patient's extensive thrombophilia workup was unremarkable.

Test	Lab values	Reference
Prothrombin time	13.7	11.7-14.5 second
Activated partial thromboplastin time	27	22.8-34.2 second
Lupus anticoagulant	Not detected	Not detected
Beta-2 glycoprotein	<2	<20 units/mL
Cardiolipin antibody	<2	<20 units/mL
Factor V Leiden mutation	Negative	Negative
Prothrombin gene mutation	Negative	Negative

**Figure 4:**
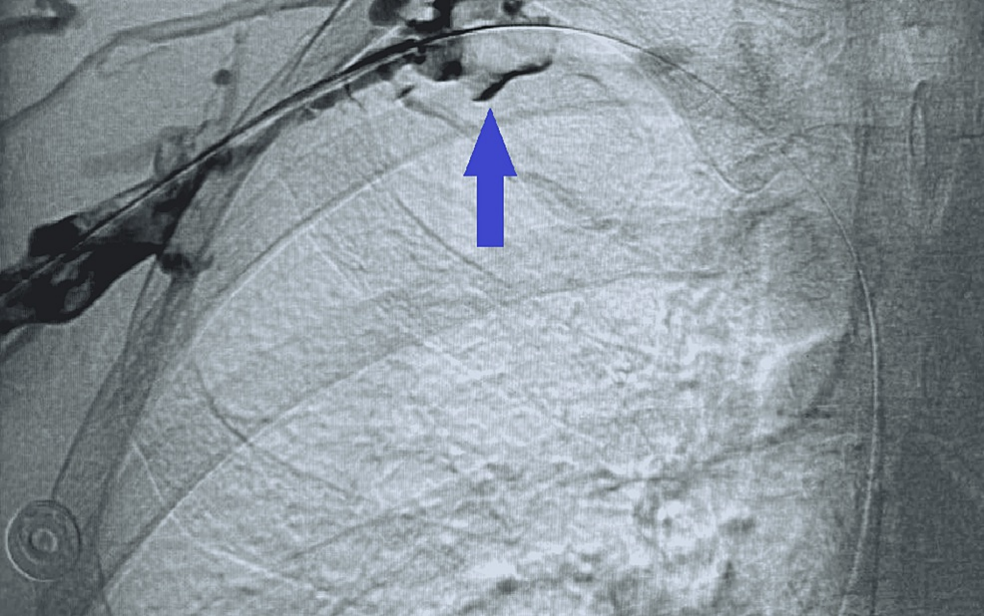
Venous contrast study showing thrombus (arrow) and flow obstruction at the thoracic outlet.

**Figure 5:**
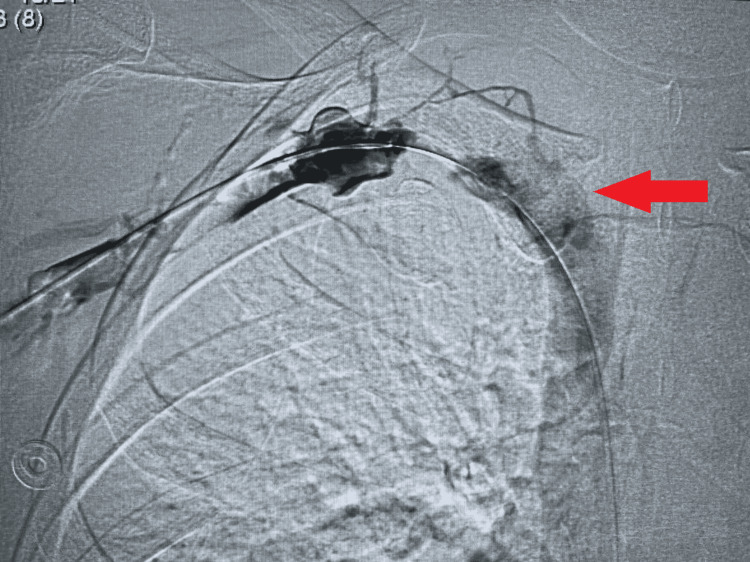
Venous contrast study showing contrast flow (arrow) after thrombolysis.

Venous duplex showed extrinsic compression of the subclavian vein as it passed under the first rib, confirmed by catheter-based contrast venography (Figures 4, 5). She underwent catheter-directed thrombolysis and subsequently was started on a therapeutic dose of 1.5 milligram/kg/day of subcutaneous enoxaparin with a plan to complete three months of anticoagulation for this provoked DVT. She was then referred to a higher center where she had a successful surgical decompression of thoracic outlet (first rib resection), improving her overall symptoms and returning to her prior functional status. 

## Discussion

Paget-Schroetter syndrome (PSS) was introduced in 1949 AD by Hughes after a compilation of numerous case reports of upper extremity deep vein thrombosis (UEDVT), which was assembled in recognition of the work by Paget in 1875 and Schroetter in 1884 where both described an association of acute venous stasis in a healthy person caused by repeated movements of shoulder girdle damaging the axillary vein, thus responsible for the formation of thrombus also known as traumatic thrombosis of the axillary vein [4,5]. This condition is often associated with the strenuous and repetitious activity of upper extremities such as wrestling, gymnastics, and swimming causing retroversion, hyperabduction, and extension of the arm, imposing excessive strain on the axillary-subclavian vein leading to microtrauma of endothelium and activation of the coagulation cascade [1]. This hypothesis is also reinforced by anatomical abnormalities involving the thoracic outlet, such as a cervical rib, congenital bands, hypertrophy of scalenus tendons, and abnormal insertion of the costoclavicular ligament in the pathogenesis of effort thrombosis [4,6]. The narrow costoclavicular space compels compression of the vein and stasis to flow, forcing restriction in the mobility of the subclavian vein and making it susceptible to trauma after recurrent arm activity. This starts a process of repetitive endothelial trauma causing intimal hyperplasia, inflammation, and fibrosis, causing venous webs, extensive collateral formation, and perivenular fibrosis, which in turn worsens stasis causing thrombus formation [1]. The key pathogenic factor is costoclavicular crowding due to anatomical abnormalities and repetitive endothelial trauma from muscle strain, causing initiation and progression of thrombosis [1]. Symptom onset is usually acute to subacute, but an infrequent patient can present with chronic symptoms, with most patients reporting a discrete precipitating event, usually sports related to arm exertion [1]. The most common symptoms are swelling, heaviness, redness, and arm discomfort [6-8]. Often, dilated and visible veins across the shoulders and upper arms (Urschel’s sign) are seen [6]. Complications include pulmonary embolism, post-thrombotic syndrome, and recurrent thrombosis [6,7]. Compression ultrasonography with a color Doppler is the preferred initial test for evaluating suspected UEDVT, whereas contrast venography is the gold standard for diagnosis [9,10]. Though venography is not required for diagnosis, it is almost always done as a part of a multimodal treatment strategy to deliver catheter-directed thrombolysis and plan for thoracic outlet decompression surgery [1,6].

Even though the role of inherited and acquired causes of thrombophilic events is unclear, it is not unreasonable to test the patients for these abnormalities as they might help predict postoperative anticoagulation [1]. The total duration of anticoagulation is unclear. However, the current standard is a minimum of three to six months of anticoagulation with low molecular unfractionated heparin or direct thrombin inhibitor [11]. Management includes both anticoagulation and catheter-directed thrombolysis. Local catheter-directed thrombolysis is recommended in all patients presenting within four and six weeks of symptom onset as the success of thrombolysis diminishes as the time from symptom increases, underscoring the need for prompt recognition and treatment [6,12]. Additionally, surgical thoracic outlet decompression, which involves resectioning the first rib, the scalene muscles, and the costoclavicular ligament, may be required [13]. In this case, as described above, the thrombus was likely secondary to the repetitive nature of the arm movements in swimming, causing the extrinsic compression of the subclavian vein when it passed under the first rib, as confirmed by catheter-based venography.

## Conclusions

PSS is a classic example of a clinical condition needing a high index of suspicion and timely diagnosis. Prompt treatment has a great outcome with minimal long-term sequelae but, if missed, is complicated by significant long-term morbidity. Therefore, physicians should be aware of this rare entity for early recognition and timely referral to vascular surgery. Further studies are required to compare the efficacy of thrombolysis with surgical options.
